# Response: Commentary: Data processing thresholds for abundance and sparsity and missed biological insights in an untargeted chemical analysis of blood specimens for exposomics

**DOI:** 10.3389/fpubh.2022.1003148

**Published:** 2022-10-18

**Authors:** Dinesh K. Barupal

**Affiliations:** Icahn School of Medicine at Mount Sinai, New York, NY, United States

**Keywords:** metabolomics, exposome, mass spectrometry, birth weight, bioinformatics

The commentary by Keski-Rahkonen et al. (1) on our study “*Data processing thresholds for abundance and sparsity and missed biological insights in an untargeted chemical analysis of blood specimens for exposomics*” (2) needed some additional comparative analyses to clarify the discrepancies noticed for the example peaks that were flagged as missed in the MS-DIAL data processing workflow used in our study. A re-evaluation of the published data matrix available at EBI Metabolights repository accession MTBLS1684 (https://www.ebi.ac.uk/metabolights/MTBLS1684/), from the original study (3, 4) indicated that the LC/MS peaks were reported by identifiers created using neutral masses and retention time (RT) pairs while assuming a proton adduct for all peaks. For example, the peak m/z 412.3035 at RT 5.75 min was reported as “X411.2972.5.749766” and the peak m/z 289.2162 at RT 4.83 min was reported as “X288.2084.4.8316393” in the submitted data matrix. However, in our study (2), MS-DIAL generated data matrix reported the observed m/z values for these peaks. Therefore, those two example peaks were flagged as being missed.

A new comparative analysis has been conducted (https://colab.research.google.com/drive/1eV2ywgLtg0RyJ9qzuVl45KGWlmD0JxPy#scrollTo=c38d63Y8pevi) to expand the discussion. Mass of proton (H+) (1.00784) was added to the reported mass of every peak in the original peak-list to make it comparable to the MS-DIAL generated peak-list. Two new Venn-diagrams (Figure 1) have been created to compare both peak-lists. Among the 623 significant peaks detected using the MS-DIAL peak list, 86% of them were not found among the significant peaks observed from the original peak list (Figure 1B and Supplementary Table S1). Only 7% of all peaks in the MS-DIAL peak list were found in the original peak-list (Figure 1A and Supplementary Table S1). These Venn-diagrams underscore the importance of careful design and review of data processing of untargeted metabolomics datasets from population-scale studies. It also strongly suggests the critical importance of cutoffs for the detection frequency and abundance parameters while generating data matrices from untargeted LC-HRMS datasets. Since MS/MS fragmentation data, cohort data, and the original unfiltered peak lists created without any thresholds for detection frequency and abundances were not made publicly available for the study, additional analyses were not feasible to expand the discussion. The current analysis is limited to unadjusted statistical results. However, the quality of raw LC-HRMS spectral data in the MTBLS1684 study is commendable, this is because of the un-noticeable retention time and signal intensity drifts for a batch of ~ 500 samples. It should be promoted as a benchmarking dataset for teaching and other analyses for comparing performances of data processing software in metabolomics and exposomics.

**Figure 1:**
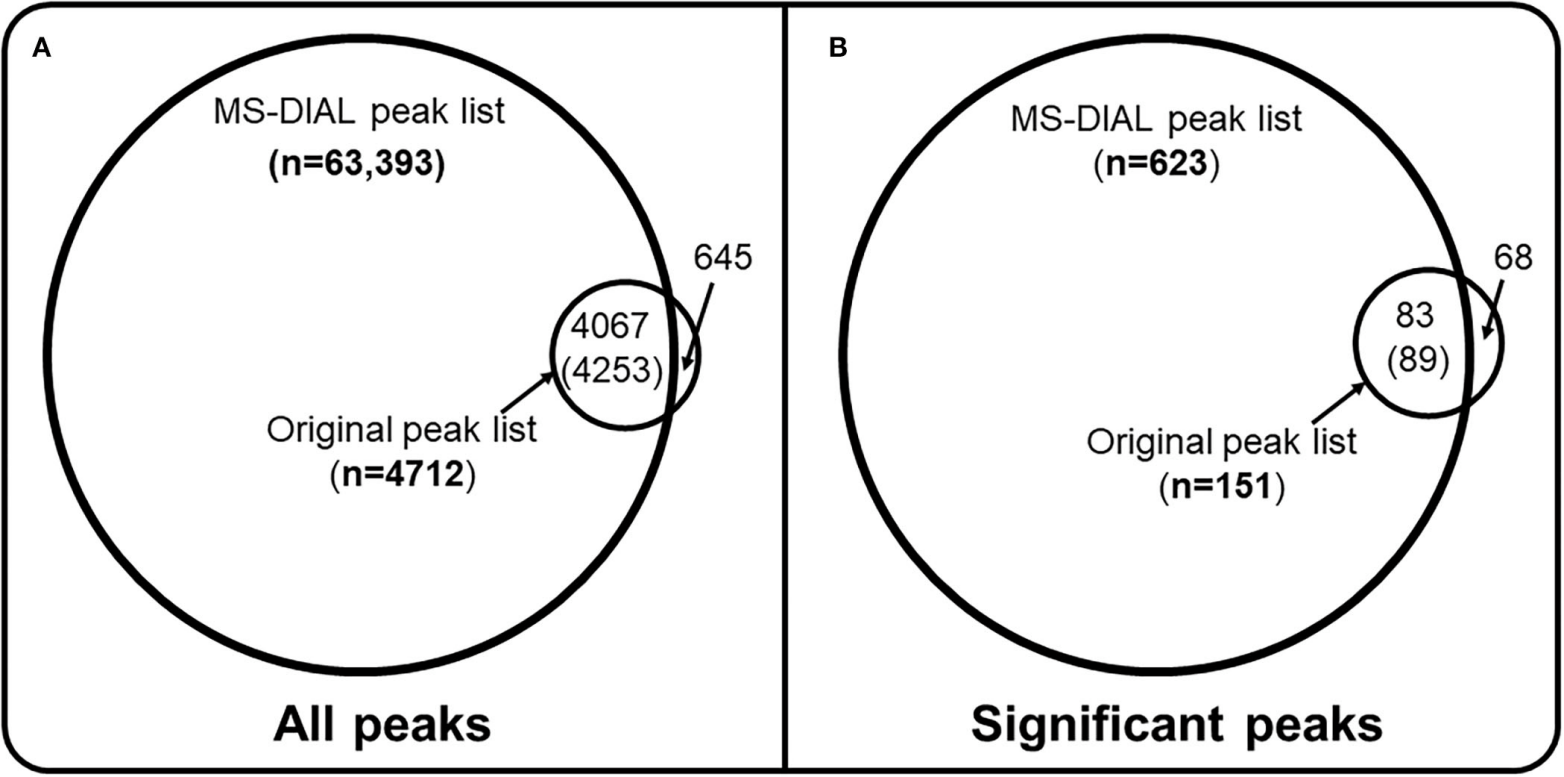
Data processing thresholds and missed biological insights. **(A)** all peaks that were generated by the MS-DIAL software for the MTBLS1684 study of 499 cord blood samples **(B)** only peaks that were found to be significantly correlated (Spearman coefficient) with birth weight at FDR threshold of 0.05. Mass of proton (1.00784) was added to the neutral mass of peaks reported in the original peak list so it could be compared against the MS-DIAL peak-list. A retention time threshold of 0.05 min and a mass accuracy threshold of 0.01 Da were used to find if a peak from the original peak list was present in the MS-DIAL generated peak list. Full code is available at https://colab.research.google.com/drive/1eV2ywgLtg0RyJ9qzuVl45KGWlmD0JxPy#scrollTo=c38d63Y8pevi. Out of 4,712 peaks from the original peak list, 645 had zero, 3,885 had one, 178 had two and four peaks had three matching peaks in the MS-DIAL data matrix, so a total of 4,253 peaks (4,253/63,393, ~7%) from the MS-DIAL data matrix were covered by the original peak list. Out of 151 significantly associated peaks **(B)** from the original peak list, 68 had 0, 77 had 1 and 6 peaks had two matching peaks among the 623 significantly associated peaks from the MS-DIAL data matrix (89/623, ~14% coverage).

## Author contributions

DB performed the data analysis and wrote the manuscript.

## Conflict of interest

The author declares that the research was conducted in the absence of any commercial or financial relationships that could be construed as a potential conflict of interest.

## Publisher's note

All claims expressed in this article are solely those of the authors and do not necessarily represent those of their affiliated organizations, or those of the publisher, the editors and the reviewers. Any product that may be evaluated in this article, or claim that may be made by its manufacturer, is not guaranteed or endorsed by the publisher.
